# Complete Genome Sequence of Goose Tembusu Virus, Isolated from Jiangnan White Geese in Jiangsu, China

**DOI:** 10.1128/genomeA.00236-12

**Published:** 2013-03-07

**Authors:** Kaikai Han, Xinmei Huang, Yin Li, Dongmin Zhao, Yuzhuo Liu, Xiaobo Zhou, Yuan You, Xingxing Xie

**Affiliations:** Institute of Veterinary Medicine, Jiangsu Academy of Agricultural Sciences, Key Laboratory of Veterinary Biological Engineering and Technology, Ministry of Agriculture, Nanjing, Jiangsu Province, China

## Abstract

Avian tembusu virus (TMUV), which was first identified in eastern China, is an emerging virus causing serious economic losses in the Chinese poultry industry. Here, we report the complete genome sequence of goose tembusu virus strain JS804, isolated from Jiangnan white geese with severe neurological signs. The genome of JS804 is 10,990 nucleotides (nt) in length and contains a single open reading frame encoding a putative polyprotein of 3,425 amino acids. Research of the whole sequence of tembusu virus will help us to understand further the molecular and evolutionary characteristics and pathogenesis of this virus.

## GENOME ANNOUNCEMENT

Since April 2010, a novel contagious disease in ducks and geese, with egg drop, feed uptake decline, and neurological signs, caused by a newly emerged virus, has spread throughout eastern China. The infection rate and morbidity of shelducks were as high as 100%, and mortality varied from 5% to 30%, possibly due to secondary bacterial infections (1). Up to now, several complete genomic sequences of duck tembusu virus (TMUV) have been reported (2–4), but there have been few reports about this virus isolated from geese.

Herein, we report a TMUV strain isolated from Jiangnan white farm geese with severe neurological signs in Jiangsu Province, China. First, in order to determine the nucleic acid type, a convenient method called DNase-SISPA (DNase-Sequence independent single primer amplification) was utilized; after that, three fragments highly homologous to the envelope (E) gene of tembusu virus were obtained and named S1, S2, and S3. Then, based on the sequence of flavivirus Bagaza virus (GenBank accession no. AY632545), 5 pairs of nucleotide primers were designed to amplify the other regions and bridge the gaps between amplified regions. At last, all of the amplified products were purified and cloned into the pMD18-T vector (TaKaRa) and sequenced in triplicate. Sequences were assembled using the SeqMan programs (DNAStar and Lasergene) to produce the final genome sequence. The isolated virus was identified as TMUV and named JS804. The full-length genome sequence of JS804 was 10,990 nucleotides (nt) in length, with a typical flavivirus genome organization, and the 5′ and 3′ untranslated regions (UTR) were 94 and 618 nt, respectively. Additionally, the coding region of JS804 included a single open reading frame (ORF) (10,278 nt) that encoded a polypeptide of 3,425 amino acids (aa), three structural proteins (capsid, prM, and envelope), and five nonstructural proteins (NS1, NS2A, NS2B, NS3, NS4A, NS4B, and NS5).

Comparison to previous avian tembusu virus strains isolated from different breeds of ducks and geese in China showed that JS804 was 99.6% identical to BYD virus (GenBank accession no. JF312912) and 99.2% identical to ZJ-6 virus (GenBank accession no. JF459991) at the nucleotide level. This indicates that all avian tembusu-related virus polyprotein sequences possess high identities at the nucleotide level. A phylogenetic tree based on the whole polyprotein sequence demonstrated that strain JS804 is more closely related to Bagaza virus, which is an African flavivirus isolate that causes disease in both humans and animals (5). Using bioinformatics tools, methyltransferase domain and ATP-binding type-1 domain were found in JS804 strain. Similarly, the potential *N*-linked glycosylation sites (NLGlys) were also found in this virus, with a distribution of two in the PrM protein, two in the E protein, three in the NS1 protein, two in the NS4B protein, and three in NS5. We found that there is one more *N*-linked glycosylation site in the E protein of JS804 than in WJ-1 (GenBank accession no. JX549382).

Therefore, the study of the whole genome sequence of goose tembusu virus profits further investigation of the epidemiology and evolution of avian tembusu virus; it also contributes to the mechanisms of virus replication and pathogenesis.

### Nucleotide sequence accession number.

The complete genome sequence of the goose tembusu virus isolate has been submitted to GenBank under the accession no. JF895923.

## References

[1] Cao Z Zhang C Liu Y Liu Y Ye W Han J Ma G Zhang D Xu F Gao X Tang Y Shi S Wan C Zhang C He B Yang M Lu X Huang Y Diao Y Ma X Zhang D 2011 Tembusu virus in ducks, China. Emerg. Infect. Dis. 17:1873–1875 22000358 10.3201/eid1710.101890 PMC3310660

[2] Su J Li S Hu X Yu X Wang Y Liu P Lu X Zhang G Hu X Liu D Li X Su W Lu H Mok NS Wang P Wang M Tian K Gao GF 2011 Duck egg-drop syndrome caused by BYD virus, a new tembusu-related flavivirus. PLoS One 6:e18106 21455312 10.1371/journal.pone.0018106 PMC3063797

[3] Yan P Zhao Y Zhang X Xu D Dai X Teng Q Yan L Zhou J Ji X Zhang S Liu G Zhou Y Kawaoka Y Tong G Li Z 2011 An infectious disease of ducks caused by a newly emerged tembusu virus strain in mainland China. Virology 417:1–8 21722935 10.1016/j.virol.2011.06.003

[4] Yun T Ye W Ni Z Zhang D Zhang C 2012 Identification and molecular characterization of a novel flavivirus isolated from Pekin ducklings in China. Vet. Microbiol. 157:311–319 22317979 10.1016/j.vetmic.2012.01.013

[5] Kuno G Chang GJ 2007 Full-length sequencing and genomic characterization of Bagaza, Kedougou, and Zika viruses. Arch. Virol. 152:687–696 17195954 10.1007/s00705-006-0903-z

